# Impact-of-COVID-19 on mortality and implications for adolescent and young-adult healthcare

**DOI:** 10.1016/j.hctj.2023.100015

**Published:** 2023-08-21

**Authors:** Yoshiyasu Takefuji

**Affiliations:** Faculty of Data Science, Musashino University, 3-3-3 Ariake Koto-ku, Tokyo 135-8181, Japan

**Keywords:** COVID-19 impact, Children and adolescent ages from 1 to 24, Mortality effects, CDC dataset

## Abstract

This paper investigates the effect of COVID-19 on mortality in the child and adolescent population of five age groups. The CDC dataset was used for calculating the number of deaths by adolescent age group from 2015 to 2020. Results showed that the older the adolescent, the stronger the effect of COVID-19 on mortality. There is no significant impact of COVID-19 on mortality of three aged groups (1–4, 5–9 and 10–14). There is a significant impact of COVID-19 on mortality of two aged groups (15–19 and 20–24). The findings indicate that effective drugs should be used for protecting them against COVID-19 and for computational ethics.

## Summary

### What is currently known?

The older the patients, the stronger the impact of COVID-19 on mortality. Elderly patients are more likely to experience severe symptoms and complications from COVID-19.

### What does this article add?

There is no significant impact of COVID-19 on mortality in people aged 14 or younger. The risk of death from COVID-19 increases with age.

### How might this information affect population research?

Age information, including for those who are 15 years old or older, is important for informing nursing practice.

## Introduction

The scope of this paper is to investigate the effect of COVID-19 on mortality in the children and adolescent population. The investigated population was divided into five age groups such as 1–4, 5–9, 10–14, 15–19, and 20–24. Centers for Disease Control and Prevention (CDC) dataset was used for the proposed investigation on mortality of youth ages from 1 to 24 years old. The Python program, children.py was developed to visualize the impact of COVID-19 on mortality rates by calculating the number of deaths in five age groups from 1 to 24 years between 2015 and 2020.^1^

According to the World Health Organization (WHO), adolescence is defined as the second decade of life from 10 to 19 years of age.^2^ Historically, adolescence typically spans from 12 to 18 years of age.^3^ The United Nations defines adolescence from 10 to 19 years of age.^4^ Adolescence is defined from 10 to 19 years of age.^5^ The Centers for Disease Control and Prevention (CDC) defines the age range for adolescents as 10–19 as and refers to 20–24-year-olds as late adolescents or young adults.^6^ The scope of this paper is to investigate impact of COVID-19 on mortality of children and adolescents from 1 to 24 years.

## Methods

The research protocol used in this paper with respect to the Declaration of Helsinki on human subject testing does not apply.

**Fig. 1:**
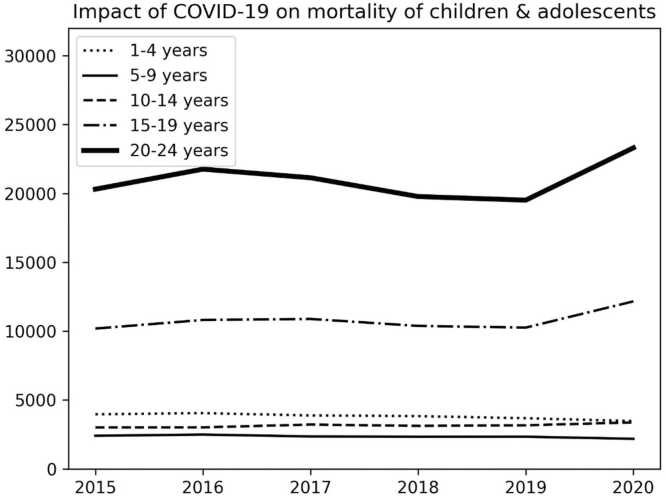
Result of adoles.py with the CDC dataset.

This paper studies the impact of COVID-19 from 2015 to 2020 on mortality of five age groups such as 1–4, 5–9, 10–14, 15–19, and 20–24. The CDC dataset^7^ was used to visualize the transition and trends of mortality from 2015 to 2020. The CDC dataset^8^ is composed of seven determinants: “Data As Of”, “Start Date”, “End Date”, “Year”, “Sex”, “Age Group and ”Total Deaths”. The Python program, children.py^1^ was developed for visualizing the number of deaths per year from 2015 to 2020 for three age groups such as 1–4, 5–9, 10–14, 15–19, and 20–24 respectively. The program automatically scrapes a dataset^8^ from the CDC site over the Internet and can extract the number of deaths in three age groups per year from 2015 to 2020 to generate three graphs as shown in Fig. 1.

## Results

In Fig. 1, the horizontal axis indicates years from 2015 to 2020. The vertical axis indicates the number of deaths by age group per year. Fig. 1 shows that the effect of COVID-19 on mortality is stronger and the number of deaths increases significantly in the older age groups. In other words, the 20–24 age group on population mortality is most affected by COVID-19, while there is no effect of the 1–14 age group on mortality. The 15–19 age group on population mortality is less affected by COVID-19. The steeper the slope of the line, the stronger the effect on adolescence mortality. The flatter the slope of the line, the weaker its influence. Fig. 1 shows that there is no significant impact of COVID-19 on mortality of three aged groups such as 1–4, 5–9, and 10–14. COVID-19 has a negative impact on mortality in two age groups: 1–4 and 5–9. Table 1 shows the detailed numbers on trends of deaths from 2015 to 2020.

**Table 1:** Trends of deaths from 2015 to 2020 by age group.

Age group	2015	2016	2017	2018	2019	2020
"1–4"	3965	4045	3880	3830	3676	3469
"5–9"	2402	2490	2354	2330	2333	2183
"10–14"	3009	3013	3217	3120	3164	3373
"15–19"	10186	10812	10886	10380	10258	12164
"20–24"	20308	21763	21139	19774	19513	23310

## Discussion

As a result of this paper, it was found that COVID-19 had a stronger effect on mortality in the older group in the five groups from 1 to 24 years. In other words, the older the age group of the adolescent, the stronger the effect on mortality due to COVID-19. The lower the age group in the adolescent, the less strong the effect on mortality. In other words, the age group of 1–14 years old has no effect by COVID-19 on mortality. COVID-19 has a negative impact on mortality in 1–4 aged group.^9^ COVID-19 is a disease that affects mortality at older ages in the adolescent.

Hunt et al. found that nearly all children with special health care needs will eventually transition to adult care. These youth were at an increased risk for poor outcomes during this transition during the pandemic, so multiple professional societies had outlined the importance of a structured transition process.^10^ This process should ensure continuous care and a smooth transition for patients and caregivers.^10^ In other words, the transfer of care from pediatric to adult health care services was a particularly vulnerable period for this age group.

In addition to the transition problem, adolescents were more likely to delay or avoid medical care than other age groups, which can lead to increased morbidity and mortality.^11^ Czeisler et al. found that adolescents aged 18–24 were the most likely to avoid medical care, followed by adults aged 25–44, 45–64, and ≥65.^11^

Ludvigsson reported that children account for 1–5% of COVID-19 cases and had milder disease than adults, with fewer deaths.^12^ Symptoms were similar to adults, but fewer children developed severe pneumonia and treatment was supportive.

This paper makes a novel contribution by demonstrating that the age of children and adolescents aged 1–24 has a significant impact on their mortality from COVID-19. There was no significant impact of COVID-19 on mortality in the three age groups of 1–4, 5–9, and 10–14. However, there was a significant impact of COVID-19 on mortality in the two age groups of 15–19 and 20–24.

Götzinger et al. found that COVID-19 is usually mild in children, but some develop severe disease that requires hospitalization and ventilation.^13^ Fatal cases are rare, but more data is needed on effective treatments. Their study highlights the importance of prevention in young people.

The results of this paper suggest a variety of possible future directions for research. Zhang et al. found that COVID-19 is a global health crisis that has disproportionately affected older adults and those with pre-existing conditions.^14^ Children are more likely to have mild cases, but a healthy diet, sufficient supply of nutrition, vaccination, and atopy may be protective factors against infection and progression of the COVID-19 disease^14^ Future work in this study could reveal factors that make COVID-19 less severe in children and young people through a detailed analysis.

## Implications and contribution

The result indicates that COVID-19 is a disease that affects mortality in the older adolescent age group. There is no impact on mortality under 14 years. COVID-19 has a negative impact on mortality in two age groups: 1–4 and 5–9. In other words, the impact on mortality is weaker the younger the age group of adolescents. This result implies that the older age group of adolescents in two age groups such as 15–19 and 20–24 needed to be protected against COVID-19 from the beginning of the pandemic. This finding indicates that there may be a link between COVID-19 mortality and metabolism.

## Ethics

This research has no fund.

## Fund

This research has no fund.

## CRediT authorship contribution statement

YT completed this research and wrote the program and this article.

## Declaration of Competing Interest

The authors declare that they have no known competing financial interests or personal relationships that could have appeared to influence the work reported in this paper.

## Data Availability

The authors do not have permission to share data.
